# The Antitumor Activity and Mechanism of a Natural Diterpenoid From *Casearia graveolens*

**DOI:** 10.3389/fonc.2021.688195

**Published:** 2021-06-25

**Authors:** Ying Li, Jun Ma, Ziteng Song, Yinan Zhao, Han Zhang, Yeling Li, Jing Xu, Yuanqiang Guo

**Affiliations:** State Key Laboratory of Medicinal Chemistry Biology, College of Pharmacy, Tianjin Key Laboratory of Molecular Drug Research, and Drug Discovery Center for Infectious Disease, Nankai University, Tianjin, China

**Keywords:** cytotoxic activity, zebrafish xenograft model, apoptosis, cell cycle, FAK-MMPs, antiangiogenesis inhibitors

## Abstract

Casearlucin A, a diterpenoid obtained from *Casearia graveolens*, has been reported to possess strong cytotoxic activity. However, the *in vivo* anti-tumor effects and the action mechanism of casearlucin A remain poorly understood. Our study revealed that casearlucin A arrested cell cycle at G0/G1 stage and induced cell apoptosis in cell level. Additionally, casearlucin A inhibited HepG2 cell migration *via* regulating a few of metastasis-related proteins. Furthermore, it inhibited tumor angiogenesis in zebrafish *in vivo*. More importantly, casearlucin A significantly inhibited cell proliferation and migration in an *in vivo* zebrafish xenograft model. Collectively, these results are valuable for the further development and application of casearlucin A as an anticancer agent.

**Graphical Abstract:**
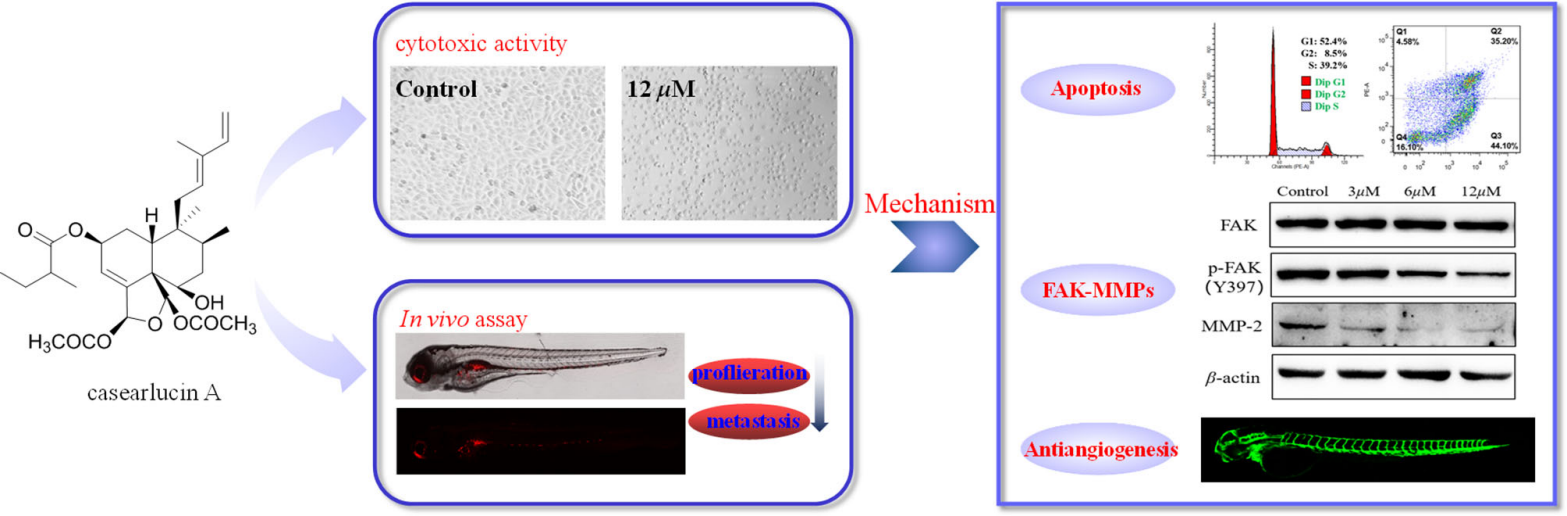


## Introduction

Cancer is the leading cause of death in the world, and the incidence rate is high in many Asian countries including China (1, 2). Nowadays, the most common ways to treat cancer are the combination of chemotherapy, radiation therapy, and surgical interventions. However, there are still many problems in current clinical treatment, such as side effects and drug resistances, which urges researchers to develop new treatment strategies or find alternative treatments (3). As we all know, medicinal plants are a promising source for developing new pharmaceuticals, and many well-known drugs approved by FDA, such as paclitaxel, camptothecin, and artemisinin, have been derived from natural products or their derivatives (4). In the development of new drugs, the main role of natural products is to provide lead compounds, or provide clues for the discovery of lead compounds.

The genus *Casearia* Jacq. belonging to the family Flacourtiaceae, consists of about 160 species, which distributed widely in tropical Africa, Asia, northwest Australia, and South America. Recent phytochemical studies have revealed the presence of abundant diterpenoids, particularly clerdoane diterpenoids, in *Casearia* plants, which showed extensive biological activities, including antimalarial, antimicrobial, antifungal, and cytotoxic activities (5–13). The plant *Casearia graveolens* evoked our interest, a phytochemical examination aiming to acquire biologically active compounds was thus carried out by our research group. In the previous chemical investigation conducted by our group, nine new clerodane diterpenoids (graveospenes A−I) and three known clerodane diterpenoids (casearlucin A, casearlucin F, and bucidarasin C) was obtained. A biological evaluation of the new compounds was performed, and graveospenes A was found to be cytotoxic (14). Therefore, three known compounds with similar structure to graveospenes A were screened for cytotoxic activity. The results revealed that all of the three compounds were cytotoxic against HepG2, A549, and Hela cell lines, and casearlucin A was the most active. According to the previous literature, there have been no reports on the mechanism of casearlucin A, especially the anti-tumor effects *in vivo*. The further research on casearlucin A with remarkable cytotoxicity may be potentially useful for the development of casearlucin A as an anticancer agent.

Casearlucin A with prominent cytotoxic activity attracted our interest, and there is no report on its action mechanism. So, this study aims to investigate the anti-tumor effects *in vivo* using a zebrafish tumor xenograft, and the anti-tumor mechanism, all of which are important and valuable for the further development and application of casearlucin A as an anticancer agent.

## Materials and Methods

### Materials and Cell Culture

Dulbecco’s modified Eagle’s medium (DMEM), Fetal bovine serum (FBS, BI, Israel) were purchased from LABBIOTECH Co. Ltd. (Shandong, China). MTT and dimethyl sulfoxide (DMSO) were purchased from Solarbio (Beijing, China). Celltracker CM-DiI was obtained from Yeasen Biotechnology Co. Ltd (Shanghai, China). Annexin V-FITC Apoptosis Detection Kit, Cell Cycle and Apoptosis Kit, BCA protein assay kit were provided by Beyotime Biotechnology Co. Ltd (Shanghai, China). Rabbit monoclonal antibodies to Bax, Bcl-2, FAK, p-FAK (phospho Y397), MMP-2, *β*-actin were all purchased from Cell Signaling Technology (Danvers, MA, USA).

The three cancer cell lines (HepG2, Hela and A549 cells) were obtained from Shanghai Institutes for Biological Sciences, Chinese Academy of Sciences (Shanghai, China). The cells were cultured in DMEM containing 10% (v/v) fetal bovine serum and 100 U/mL penicillin/streptomycin under a water-saturated atmosphere of 95% air and 5% CO_2_.

### Cytotoxic Activity Assay

The cytotoxic activities were evaluated using MTT assay (15). Briefly, after reaching approximate 80% confluence, the cells were harvested and seeded in 96-well plates (1×10^4^ cells/well) and allowed to adhere for 24 h at 37°C. Then, the cells were treated with the test samples dissolved in DMSO at different concentrations, including the positive. Etoposide was used as a positive control. After continuous incubation for 48 h, 20 *μ*L MTT solution (5 mg/mL) was added in each well for 4 h incubation. Then, the medium was replaced with 150 *μ*L DMSO and the absorbance was measured at 492 nm using microplate reader (Thermo Fisher Scientific Inc. America). The experiments were performed in triplicate, and the IC_50_ value was defined as the concentration of the compounds that inhibited cell proliferation by 50%.

### Wound-Scratch Assay

The effects of casearlucin A on motility ability of HepG2 cells were assessed using wound scratch assay (16). Briefly, cells were seeded into 6-wells plate with a concentration of 5 × 10^5^cells/well. After reaching 90% confluence, cells were scratched by a sterile pipette tip. Then, the cells were treated with various concentrations of casearlucin A. The wounded healing was observed using microscope at 0 and 48 h. The scratch distance value was measured by Image J software.

### Cell Cycle Analysis

The distribution of cell cycle of HepG2 cells affected by casearlucin A was performed using Flow cytometric analysis (17). HepG2 cells in exponential growth phase were seeded in 12 well plate at the density of 2 × 10^5^ cells/well for 24 h at 37 °C. Then, the cells were treated with different concentrations of casearlucin A (2, 4, and 8 *μ*M). After an exposure to the test sample for 48 h, the cells were harvested, washed with PBS twice, and fixed in 70% ice-cold ethanol at 4°C overnight. Then, the cells were washed with PBS twice and treated with propidium iodide staining buffer containing RNase (Beyotime, C1052) for 30 minutes at 37°C in the dark, followed immediately by cellar DNA analysis using BDLSR Fortessa flow cytometry. Data were processed using ModFit LT Software.

### Apoptosis Analysis by Flow Cytometry

The apoptosis analysis of HepG2 cells induced by the tested compound was accomplished by flow cytometry using Annexin V-FITC Apoptosis Detection Kit (Beyotime, C1062L) according to the manufacturer’s instructions (18, 19). Briefly, HepG2 cells were harvested and seeded in 12-well plates (1 × 10^5^ cells/well) and allowed to adhere for 24 h at 37°C. Then, the cells were treated with various concentrations (5, 10, and 15 *μ*M) of casearlucin A. After 48 h incubation, the cells were washed twice with PBS and resuspended in the binding buffer (Beyotime, Shanghai, China). This suspension was incubated for 20 min at room temperature in the dark after adding 10 *μ*L Annexin V-FITC and 5 *μ*L PI. Then, cell apoptosis was examined by BD LSRFortessa flow cytometry (BD Biosciences). The cell apoptosis data were obtained with FLOWJO flow cytometry analysis software (FLOWJO LLC, Ashland, OR, USA).

### Western Blot Analysis

HepG2 cells were seeded in 6-well plate at the density of 1 × 10^5^ cells/well for 24 h. Then, the cells were treated with casearlucin A for 36 h, the cells were washed with cold PBS twice and collected. The cells were lysed with lysis buffer containing protease inhibitor cocktail and PMSF. Then, the lysates were centrifuged at 10,000 rpm for 10 min and the supernatants were collected to acquire the total protein. Protein concentrations were quantified using the BCA protein assay kit (Beyotime, P0012S). The proteins were separated by 10% SDS-PAGE and transferred to polyvinylidene difluoride. The membrane was blocked with 5% skim milk for 1 h at room temperature and then incubated (4°C, overnight) with primary antibodies against Bax (Cell Signaling Technology, 14796S), Bcl-2 (Cell Signaling Technology, 4223S), FAK (Cell Signaling Technology, 3285S), p-FAK (phospho Y397) (Cell Signaling Technology, 8556S), and MMP-2 (Cell Signaling Technology, 87809S). The membranes were subsequently incubated with horseradish peroxidase-conjugated secondary antibody for 1 h at room temperature. Lastly, the protein blots were visualized using an ECL detection kit (Beyotime, P0018AS). *β*-Actin protein (Cell Signaling Technology, 4970S)was used as an internal reference. Each band was quantified by Image J software (20).

### Toxicity Screening of Zebrafish Embryos

Adult AB strain zebrafish were obtained from School of Medicine, Nankai University (Tianjin, China). Embryos were taken from adult zebrafish, which were placed in the breeding room overnight and mixed for 30 min to give fertilized eggs. Embryos were cultured in Holt buffer (NaCl 59.9 mM, KCl 0.7 mM, NaHCO_3_ 0.3 mM, and CaCl_2_ 0.9 mM) for further experiments.

The healthy zebrafish embryos were selected and treated with casearlucin A of 2.5, 5, 10, and 20 *µ*M, to assess the toxicity of the compound on developing zebrafish embryos.The deformity rate and mortality rate were recorded every 24 h after continuous administration. All the procedures involving animals were approved by the Institutional Animal Care Committee of Nankai University (No. SYXK (JIN) 2019-0001).

### Antiangiogenetic Assay Using Transgenic Zebrafish Model

The angiogenesis inhibitory activity of the selected compound was carried out using transgenic zebrafish *Tg (fli1: EGFP)*. Transgenic zebrafish were obtained from Shanghai FishBio Co., Ltd. The embryos were obtained from adult Tg zebrafish as reported previously (21, 22). Briefly, 6 hour post fertilization (hpf) embryos were grouped randomly and placed in the 24-well plate, and then the embryos were exposed to various concentrations of casearlucin A for 48 h at 28.5°C. After the treatment, embryos were anesthetized with 0.02% tricaine and photographed by a confocal microscopy (Leica, Germany). The development of intersegmental blood vessels (ISVs) and dorsal longitudinal anastomotic vessels (DLAVs) at 48 hpf were observed, and the length of ISV vessels was measured using Image J software.

### *In Vivo* Anti-Tumor Assay Using Zebrafish Model

Embryos were obtained from adult AB zebrafish as reported previously (23). 48 hours post-fertilization (hpf) embryos were utilized to establish a xenograft tumor model (24). Prior to microinjection, HepG2 cells were labeled with 2 *μ*M CM-DiI (Yeasen, 40718ES50). Then, the cells were resuspended in serum-free DMEM medium and adjusted to a density of 1 × 10^7^ cells/mL. Subsequently, 48 hpf embryos were anesthetized and microinjected into the yolk with 5 nL stained cells. After 4 h incubation, tumor-bearing embryos were randomly divided into five groups (15/group) and treated with different concentrations of casearlucin A by soaking. The embryos were incubated continuously for 48 h at 28.5°C. Lastly, images were captured at 5 days post-fertilization using a confocal microscopy (Leica, Germany), and the density and focus number of red fluorescence was measured using Image J software.

### Statistical Analysis

Data were analyzed by GraphPad Prism and presented as mean ± SD. Probabilities (P) less than 0.05 were determined to be significant by analysis of variance (ANOVA). The differences among three or more groups were analyzed by one-way ANOVA multiple comparisons. The experiments were repeated three times.

## Results

### Casearlucin A Inhibited Cancer Cell Growth *In Vitro*

**Figure 1:**
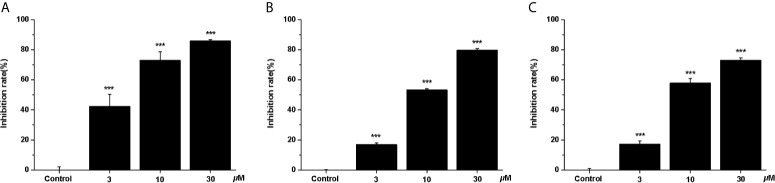
Anti-proliferative effects of casearlucin A treatment on three cell lines. HepG2 **(A)**, A549 **(B)** and Hela **(C)** cells were treated with casearlucin A (3, 10, and 30 *μ*M) for 48 h. Cell viability was examined using MTT assay. DMSO was used as a negative control. The results are presented as means ± SD. ***p < 0.001 *versus* control group.

In recent years, the potential therapeutic effects of bioactive natural products for cancer treatment have attracted broad attention (4). Thus, we studied the anti-cancer effects of three clerodane diterpenoids (casearlucin A, casearlucin F and bucidarasin C) from *Casearia graveolens*. To assess the anti-proliferative activity of the compound, three human cancer cell lines (HepG2, A549, and Hela cells) were selected to test their toxicities by MTT assays (25, 26). Etoposide was used as a positive control (27, 28). Results showed that casearlucin A was the most active and reduced the cell growth in a dose-dependent manner (**Figure 1**). Their respective IC_50_ values on three cell lines were calculated and shown in **Table 1**, casearlucin A intensively inhibited the three tumor cell lines with lower IC_50_ values than etoposide, especially on HepG2 cells.

**Table 1:** Cytotoxicities of three compounds against three human cancer cell lines.

Compound	HepG2 (IC_50_, *μ*M)	A549 (IC_50_, *μ*M)	Hela (IC_50_, *μ*M)
Casearlucin A	3.8 ± 0.3	9.9 ± 0.1	10.5 ± 0.8
Casearlucin F	9.1 ± 0.9	17.1 ± 1.8	15.6 ± 1.1
Bucidarasin C	10.2 ± 1.0	16.6 ± 0.7	7.2 ± 0.2
Etoposide^a^	15.7 ± 1.6	42.7 ± 2.7	31.0 ± 0.7

a Etoposide was used as a positive control. All results are expressed as mean ± SD.

**Figure 2:**
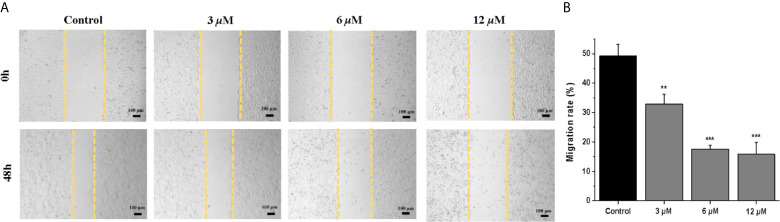
Casearlucin A inhibited HepG2 cells in wounded healing assay. **(A)** HepG2 cells were photographed at 0 h and 48 h (scale bar: 100 *μ*m). Data of Migration rate (%) were shown in **(B)**. DMSO was used as a negative control. The results are presented as means ± SD. **p < 0.01, and ***p < 0.001 *versus* control group.

Next, we further investigated the inhibitory effects of casearlucin A on cell migration, the wounded healing assay was performed (29, 30). As shown in **Figure 2**, after 48 h treatment of different concentrations of casearlucin A, the migration of HepG2 cells were markedly inhibited.

### Effects of Casearlucin A on Cell Cycle

**Figure 3:**
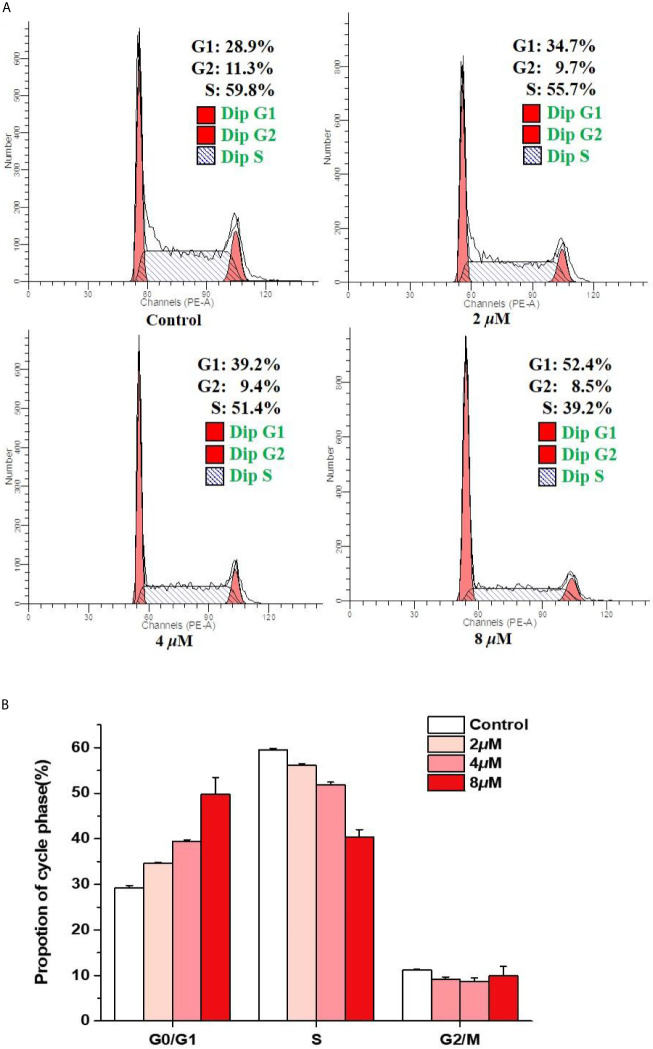
Arrest effects of casearlucin A on HepG2 cell cycle. HepG2 cells were treated with different concentrations (2, 4, and 8 *μ*M) of casearlucin A for 48 h. DMSO was used as a negative control. **(A)** The cells were harvested and stained with propidium iodide (PI), and the cell cycle distribution was analyzed using flow cytometry. **(B)** Data processing of cell cycle distribution. Data from three separate experiments are expressed as means ± SD.

In order to investigate whether casearlucin A inhibited the growth of HepG2 cells by regulating the cell cycle, we first detected its contribution to the induction of cell cycle arrest by flow cytometric analysis (31, 32). HepG2 cells were treated with various concentrations of casearlucin A and then were stained with PI for cell cycle analysis. With the concentrations of casearlucin A increasing from 2 *μ*M to 8 *μ*M, the percentage of cells in the G1 phase increased from 28.9% (control) to 34.7% (2 *μ*M), 39.2% (4 *μ*M), and 52.4% (8 *μ*M), while the S and G2 phase decreased after treated with different concentrations (**Figure 3**). Collectively, these results implied that casearlucin A could induce G0/G1 cell cycle arrest in HepG2 cells.

### Apoptosis Effects Induced by Casearlucin A

**Figure 4:**
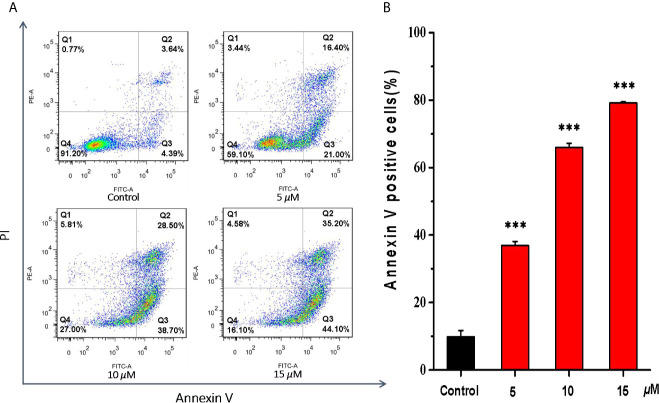
Apoptosis effects of HepG2 cells induced by casearlucin A. HepG2 cells were treated with different concentrations (5, 10, and 15 *μ*M) of casearlucin A for 48 h. DMSO was used as a negative control. Then, the cells were harvested, stained with Annexin V and propidium iodide (PI), and subsequently analyzed by flow cytometry. **(A)** Flow cytometric analysis of HepG2 cells after treated with different concentrations of casearlucin A. **(B)** Histogram of apoptotic cells at 48 h with the treatment of casearlucin A. Data from three separate experiments are expressed as means ± SD. ***p < 0.001 *versus* control group.

Then, the effects of casearlucin A on cell apoptosis were investigated in HepG2 cells. We analyzed the apoptosis by flow cytometry using Annexin V/PI double staining (33, 34). As shown in **Figure 4**, we found that the population of the early and late apoptotic cells increased dramatically. With the increase of concentrations of casearlucin A, the percentage of apoptotic cells rose from 8.0% (control) to 37.4% (5 *μ*M), 67.2% (10 *μ*M) and 79.3% (15 *μ*M). The data indicated that the apoptosis of HepG2 cells induced by casearlucin A was dose-dependent. As a result, casearlucin A was proven to possess strong apoptotic induction abilities against HepG2 cells.

**Figure 5:**
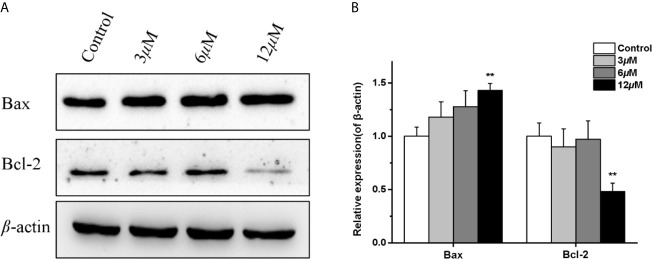
Effects of casearlucin A on apoptosis related proteins expression in HepG2 cells. HepG2 cells were pre-treated with casearlucin A for 36 h, and western blotting analysis was performed. DMSO was used as a negative control. **(A)** Western blotting results of protein levels. **(B)** Quantitative analysis of apoptosis related proteins expression. *β*-Actin protein was used as internal reference. **p < 0.01 compared with control group cells. Data were obtained by at least three independent experiments.

Moreover, since the Bcl-2 family proteins are the key regulators of mitochondrial membrane function, which have pivotal roles in the regulation of mitochondrial apoptosis. We examined the effects of casearlucin A on the expression of anti-apoptotic Bcl-2 protein, and pro-apoptotic Bax protein. As the results illustrated in **Figure 5**, casearlucin A treatment increased the protein levels of Bax, and decreased the protein levels of Bcl-2.

### Casearlucin A Inhibited HepG2 Cell Metastasis *via* Regulating FAK/MMPs Signaling Pathway

**Figure 6:**
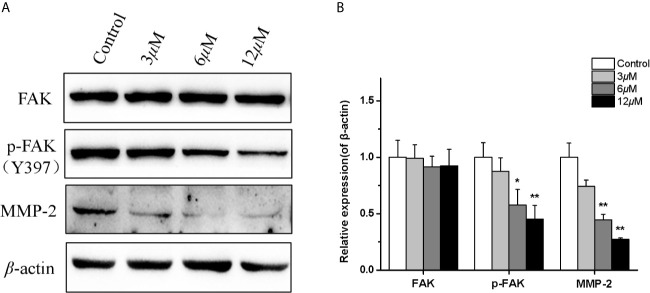
Effects of casearlucin A on metastasis-related proteins expression in HepG2 cells. HepG2 cells were pre-treated with casearlucin A for 36 h, and western blotting analysis was performed. DMSO was used as a negative control. **(A)** Western blotting results of protein levels. **(B)** Quantitative analysis of metastasis-related proteins expression. *β*-Actin protein was used as an internal reference. *p < 0.05, and **p < 0.01 compared with control group cells. Data were obtained by at least three independent experiments.

In order to further reveal the mechanism of casearlucin A inhibiting cell metastasis, a few of metastasis-related proteins were detected after 36 h treatment (35, 36). Many studies have reported that matrix-metalloproteinases (MMPs) play key roles in tumor progression and metastasis, tumor cells usually have high expression levels of MMP. Our results revealed that the protein expression level of MMP-2 in HepG2 cells was obviously reduced in a dose-dependent manner after casearlucin A treatment (**Figure 6**). Then, the mechanism underlying casearlucin A-induced downregulation of MMP-2 expression in HepG2 cells was investigated, and accumulating evidence have demonstrated that the phosphorylation at Tyr 397 of FAK could promote the combination between FAK and PI3K, which in turn activated Akt, ultimately increased the MMPs. Thus, as shown in **Figure 6**, we further confirmed that the phosphorylation of FAK was decreased when HepG2 cells were treated with casearlucin A.

### Toxicity Screening of Casearlucin A to Zebrafish Embryos

**Figure 7:**
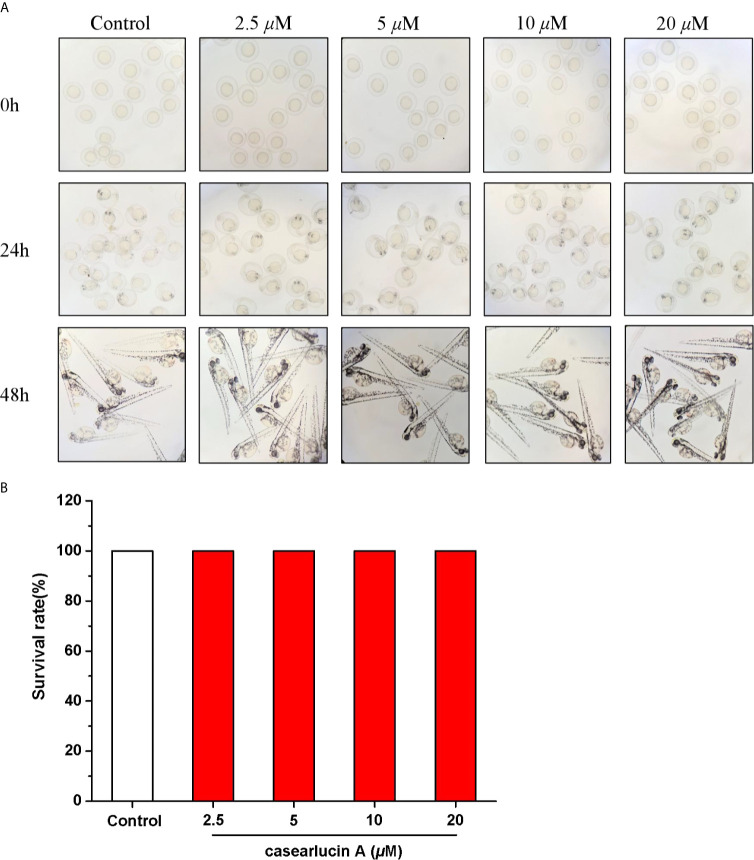
Developmental toxicity of casearlucin A in Zebrafish Embryos. The zebrafish embryos were treated with casearlucin A (2.5, 5, 10, and 20 *μ*M) for 48 h. **(A)** The morphology of zebrafish. **(B)** The survival rate of zebrafish. DMSO was used as a negative control.

The zebrafish embryos were treated with casearlucin A (2.5 to 20 *µ*M) for 48 h. As shown in **Figure 7**, the morphology of zebrafish was healthy without deformity, tail dysplasia, and yolk enlargement under the concentration of 2.5−20 *µ*M. Hence, there was no toxicity to zebrafish after the treatment of casearlucin A (2.5−20 *µ*M). Based on the results, the concentrations of 5, 10, 20 *µ*M were selected for further *in vivo* research.

### Antiangiogenetic Activity of Casearlucin A Using a Transgenic Zebrafish Model

**Figure 8:**
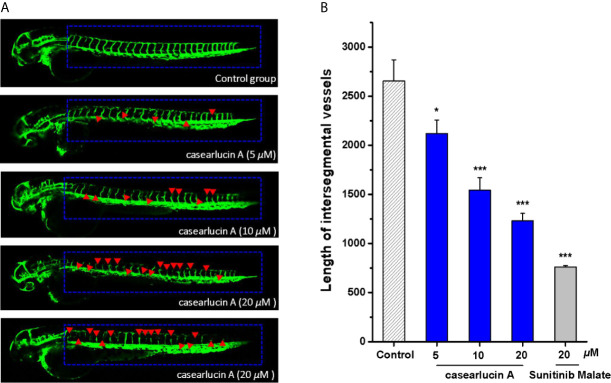
Anti-angiogenesis activity of casearlucin A in transgenic zebrafish model. The embryos from transgenic zebrafish *Tg (fli1: EGFP)* were treated by the tested compound and the anti-angiogenetic compound, sunitinib malate (positive control). DMSO was used as a negative control. After exposure to the compounds for 48 h, the development of intersegmental vessels (ISVs) and dorsal longitudinal anastomotic vessels (DLAVs) were observed, and the length of ISV vessels was measured using ImageJ program. **(A)** Representative images of zebrafish embryos treated with vehicle, casearlucin A, and sunitinib malate. **(B)** The average length of ISVs of zebrafish after treated with different concentrations of casearlucin A (5, 10, and 20 *μ*M). (n = 15 for each experimental group). *p < 0.5, ***p < 0.001 *versus* control group.

In this study, the effects of casearlucin A on zebrafish intersegmental vessels formation were examined. Herein, we used the transgenic zebrafish embryos to directly visualize the effect of casearlucin A on the new vessel development (37, 38). As shown in **Figure 8**, in contrast with the blank control, the intersegmental vessels (ISVs) and dorsal longitudinal anastomotic vessels (DLAVs) were absent and broken after treatment with casearlucin A and the positive control, sunitinib malate. According to the satanical results, the average length of ISVs of the control group was 2654.9 *μ*m, and the length decreased in a dose-dependent manner (2119.0 ± 137.7 *μ*m at 5 *μ*M, 1542.6 ± 126.4 *μ*m at 10 *μ*M, and 1232.0 ± 74.6 *μ*m at 20 *μ*M) with the increase of concentrations of casearlucin A.

### *In Vivo* Anti-Tumor Activity of Casearlucin A Using a Zebrafish Model

**Figure 9:**
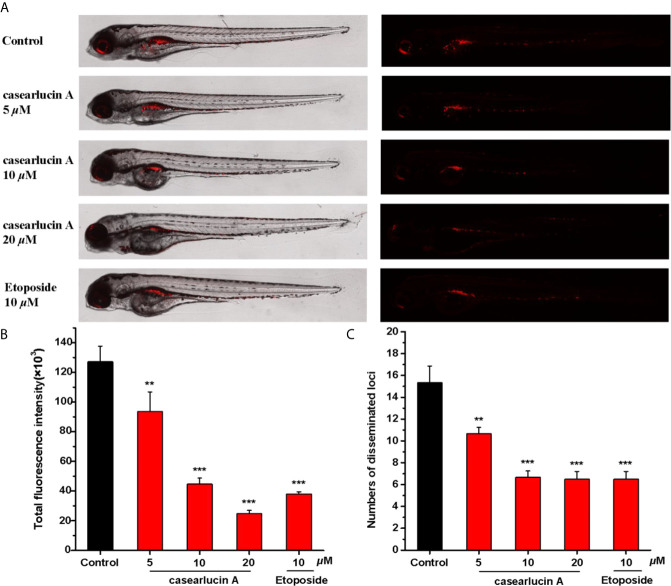
*In vivo* anti-tumor effects of casearlucin A in zebrafish xenografts. CM-DiI stained HepG2 cells were transplanted into 2 dpf zebrafish embryos by microinjecting. 4 h later, tumor-bearing embryos were treated with casearlucin A (5, 10, and 20 *μ*M) and etoposide (10 *μ*M) for 48 h (n = 15/group). DMSO was used as a negative control. **(A)**Intensity and distribution of the red fluorescence were imaged under a confocal microscope. **(B)** Fluorescence intensity of the tumor xenografts, representing the number of HepG2 cells. **(C)** Quantification of the fluorescent area of the tumor xenografts, representing HepG2 cell metastasis. Results are expressed as means ± SD. **p < 0.01 and ***p < 0.001 *versus* control group.

Previous studies showed that casearlucin A exhibited cytotoxic activity to three human tumor cells *in vitro*, and markedly inhibited the migration of HepG2 cells. Thus, we examined whether the compound could block HepG2 cells proliferation in zebrafish xenograft tumor model (39, 40). HepG2 cells labeled with CM-DiI were microinjected into the yolk sac of 48 hpf embryos, and examined by fluorescence microscopy. As presented in **Figure 9**, treatment with casearlucin A significantly decreased cell proliferation and migration, and the compound showed inhibition effects in dose-dependent manners. The results showed that casearlucin A effectively blocked tumor cell invasion and metastasis, which was comparable to the positive control, etoposide.

## Conclusion and Discussion

As numerous studies reported, because of its abnormal proliferation and high invasiveness, the treatment of cancer is very difficult. A key feature of tumor proliferation and metastasis processes is abnormal angiogenesis. Thus, the therapeutic agents targeting these characteristics will be more effective.

In our study, we have taken out a series of experiments to detect the effects of casearlucin A on cancer cell proliferation and migration *in vitro*. The results showed that casearlucin A exhibited the cytotoxicity against the selected cancer cell lines and possessed the most cytotoxic effects against HepG2 cells with an IC_50_ value of 3.8 *μ*M. Meanwhile, the wound healing assay indicated that HepG2 cells showed a migration inhibition tendency after treated by casearlucin A. Furthermore, we used zebrafish as a powerful and reliable preclinical animal model to further study the inhibitory activity of casearlucin A on the proliferation and metastasis of HepG2 cells, which revealed the anti-tumor activity *in vivo* of casearlucin A. Taken together, the *in vitro* and *in vivo* promising results suggested that the mechanism of casearlucin A should be further assessed.

The subsequent preliminary mechanism investigation revealed that casearlucin A induced the apoptosis and arrested the HepG2 cell cycle at the G0/G1 stage to exert cytotoxic effects. Moreover, we examined the expression levels of the representative proteins associated with cell apoptosis. It has been reported that the proteins of Bcl-2 family play a vital role in the mitochondria-mediated apoptosis pathway. In our study, the inhibition of Bcl-2 expression and the increase in Bax protein level by casearlucin A, demonstrated that the compound could promote apoptosis by targeting mitochondrial pathway.

In addition, FAK/MMPs signaling pathway had been proved to play critical roles in cancer cell migration and invasion. As we all know,, over-expressed FAK protein participates in the formation of focal adhesions and activates signaling pathways related to proliferation, cell migration, and angiogenesis. Furthermore, many studies have reported that matrix-metalloproteinases (MMPs) are overexpressed in almost all cancer types of cancer, and play key roles in the cancer progression and aggression (41, 42). While, as a major kinase of focal adhesion, in this process, the phosphorylation at Tyr 397 of FAK could promote the combination between FAK and PI3K, ultimately increasing the MMPs (43). Our results revealed that casearlucin A treatment decreased the phosphorylation of FAK, and the protein expression level of MMP-2 was obviously reduced in a dose-dependent manner.

Moreover, the abnormal angiogenesis is a key feature of tumor proliferation and metastasis, and thus, angiogenesis is considered as an attractive target for cancer therapeutic strategy. In our study, we used the transgenic zebrafish embryos to directly demonstrate its anti-angiogenic *Tg (fli1: EGFP)* activity.

For a long time, the favorable efficacy and low side effect of phytochemicals promoted people to seek for anti-cancer drug candidates from natural source. In our study, casearlucin A had stronger cytotoxic activity than the positive control etoposide, and there was no toxicity to zebrafish embryo development under the concentration of 2.5−20 *µ*M. With the in-depth research, the potential pharmacological effects and mechanism had been evaluated. In conclusion, casearlucin A may be a novel chemotherapeutic drug for cancer by inhibiting cell proliferation and migration and blocking angiogenesis.

## Data Availability Statement

The original contributions presented in the study are included in the article/supplementary material. Further inquiries can be directed to the corresponding authors.

## Ethics Statement

The animal study was reviewed and approved by Experimental animal ethics committee of Nankai University.

## Author Contributions

JX & YG: Designing the experiments and directing the study. YiL: Performing the experiments, analyzing the data, and writing the original draft preparation. JM: Performing the extraction, purification, and structure identification of casearlucin A. ZS, YZ, HZ & YeL: Reviewing and revising the manuscript. All authors contributed to the article and approved the submitted version.

## Funding

This research was supported financially by the National Natural Science Foundation of China (Nos. 22077067 and U1801288), the Natural Science Foundation of Tianjin, China (No. 19JCYBJC28100), Hundred Young Academic Leaders Program of Nankai University, and the Fundamental Research Funds for the Central Universities, Nankai University (No. 63201236).

## Conflict of Interest

The authors declare that the research was conducted in the absence of any commercial or financial relationships that could be construed as a potential conflict of interest.
